# Small Change: Study of Enhancer Supports Evolution Model

**DOI:** 10.1371/journal.pbio.0040419

**Published:** 2006-11-14

**Authors:** Richard Robinson

To the layperson, one fruit fly may look pretty much like another. But there are, in fact, nearly 4,000 distinct species, differing in such observable characteristics as the pattern of veins on the wings or the position of bristles on the thorax. These genetically controlled differences have evolved over millions of years, since the first ancestral fruit fly arose. And it’s not just flies, of course—every species has evolved a unique form, correlated with subtle but telling differences in their genes.

Despite the ubiquity of this phenomenon, the exact genetic underpinnings of the morphological differences between two closely related species are known in very few cases. In a new study, Sylvain Marcellini and Pat Simpson explore the sequences controlling the minutest of differences between two fruit fly species and show that the appearance of two versus four thoracic bristles is due to small sequence changes in the regulatory region of a single gene shared between the two species.

Adult flies bear numerous mechanosensory hairs, or bristles, in rows along the back of the thorax. *Drosophila melanogaster* bears two such bristles, while *D. quadrilineata* bears four. Bristle formation is due to expression of the *scute* gene, which is under the control of numerous genetic sequences, including so-called enhancers—nearby DNA sequences that bind transcription factors and enhance gene expression. One, called the dorsocentral enhancer (DCE), has been well characterized in flies and is known to interact with a transcription factor called Pannier.

**Figure:**
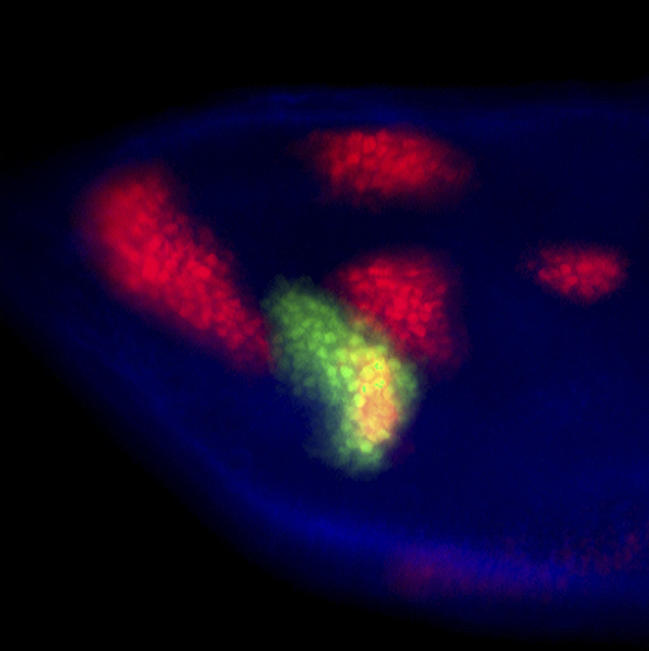
In the wing discs of *Drosophila* species, the divergent activities of proneural enhancers (orange versus green nuclei) contribute to the evolution of the adult bristle patterns.

When the authors compared the sequences of the DCEs of *D. quadrilineata* and *D. melanogaster*, they discovered that both contained binding sites for Pannier, but the sequences were significantly different in other respects, consistent with the 60 million years of evolution separating them. By staining developing flies of both species to reveal *scute* expression, they showed that the *D. quadrilineata scute* is expressed more anteriorly than the *D. melanogaster scute*, in exactly the locations that later sprout the extra bristles. When the authors inserted the *D. quadrilineata* DCE into *D. melanogaster*, the *scute* gene was active more anteriorly, and the flies developed four, instead of two, bristles, mimicking the phenotype of the *D. quadrilineata* fly. Importantly, this effect could not be reproduced when the control DCE from a different two-bristle fly was inserted.

These results provide evidence for a common model of morphologic evolution, in which slight changes in enhancers lead to slight changes in the expression domains of specific genes, leading to slight changes in the phenotype of the organism. Such small individual changes don’t necessarily alone lead to speciation, but the accumulation of such differences, combined with and reinforcing the behavioral isolation of two diverging groups, may result in the creation of a new species. While this model is widely accepted, actual examples of enhancer-driven phenotypic differences have been scarce, and so these results provide important evidence to strengthen it.

